# Supplementary material for: Identification of photoperiod-induced specific miRNAs in the adrenal glands of Sunite sheep (Ovis aries)
Source: Front Vet Sci. 2022 Jul 22;9:888207. doi: 10.3389/fvets.2022.888207 (PMC9354845; doi:10.3389/fvets.2022.888207)
Supplement: Supplementary file 1 [file Data_Sheet_1.ZIP › Supplementary file/miRDeep2 file.zip!/miRDeep2 file/Novel_Structure/SPLP42A3/Novel_624.pdf]

Provisional ID	:	NC_019459.2_6879
Score total	:	2.1
Score for star read(s)	:	-1.3
Score for read counts	:	-0.3
Score for mfe	:	2.2
Score for randfold	:	1.6
Score for cons. seed	:	
Total read count	:	11
Mature read count	:	11
Loop read count	:	0
Star read count	:	0

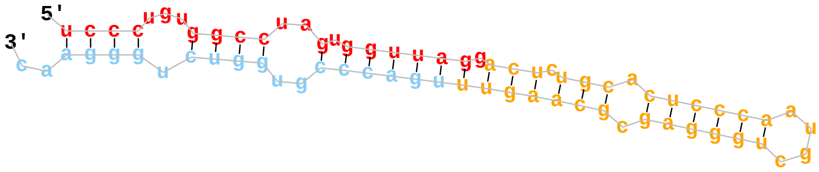

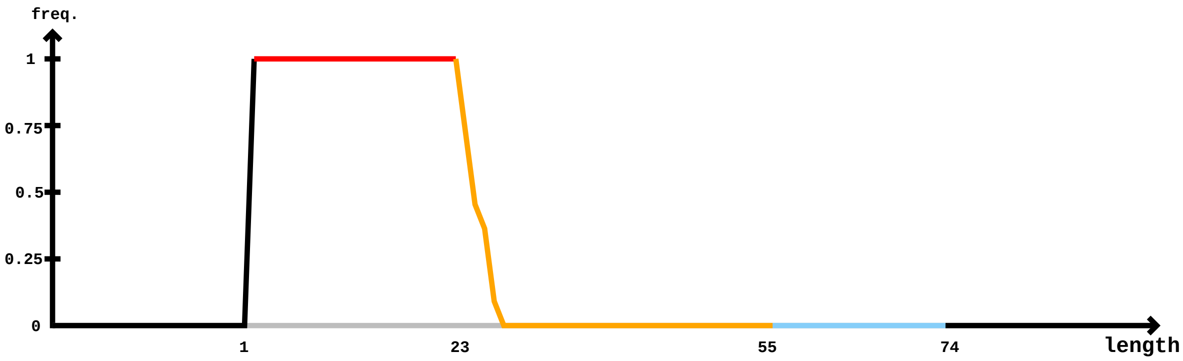

Mature **Star**

```
5'- ugaaagagguugugggacuucccuguggccuagugguuaggacucugcacucccaaugcugggagcgcaaguuugacccguggucugggaaccaagaucccacaugccgcaca   -3'    exp
    .....(.((((((((((((((((...((((..(.((((((.(((.(((.((((((....)))))).)))))))))))))..)))).))))....)).))))))).))).)...    reads   mm   sample
    ...................ucccuguggUcuagugguuagg........................................................................    4       1    seq
    ...................ucccuguggUcuagugguuagga.......................................................................    2       1    seq
    ...................ucccuguggUcuagugguuaggac......................................................................    1       1    seq
    ...................ucccuguggUcuagugguuaggacu.....................................................................    3       1    seq
    ...................ucccuguggUcuagugguuaggacuc....................................................................    1       1    seq
```
